# Impact Testing in Implant-Supported Prostheses and Natural Teeth: A Systematic Review of Properties and Performance

**DOI:** 10.3390/ma17164040

**Published:** 2024-08-14

**Authors:** Jordi Martí-Vigil, Joan Casamitjana, Xavier Marimon, Miguel Cerrolaza, Raul Medina-Gálvez, Oriol Cantó-Navés, Miquel Ferrer, Josep Cabratosa-Termes

**Affiliations:** 1 School of Dentistry, Universitat Internacional de Catalunya (UIC), 08017 Barcelona, Spain; od093977@uic.es (J.M.-V.); ruldoc@uic.es (R.M.-G.); cabratosa@uic.es (J.C.-T.); 2 Universitat Pompeu Fabra (UPF), 08002 Barcelona, Spain; joancasamitj@gmail.com; 3 Bioengineering Institute of Technology, Universitat Internacional de Catalunya (UIC), 08017 Barcelona, Spain; 4 School of Engineering, Science & Technology, Valencian International University, 46002 Valencia, Spain; miguelenrique.cerrolaza@professor.universidadviu.com; 5 Department of Strength of Materials and Structural Engineering, Universitat Politècnica de Catalunya (UPC-BarcelonaTECH), 08034 Barcelona, Spain; miquel.ferrer@upc.edu

**Keywords:** impact test, dynamic force, dynamic load, dental implant, rehabilitation materials, stress distribution

## Abstract

Dental implants offer an effective solution for partial and total edentulism, but mechanical and biological complications exist. Furthermore, high occlusal loads challenge implants and lead to potential failures. This review focuses on impact testing in contrast to incremental and static tests, an underexplored aspect of assessing daily loads on implants, bringing to light potential complications. The review examines studies employing impact forces to assess implant-supported prostheses and natural teeth properties, highlighting their significance in dental research. A systematic search following PRISMA guidelines identified 21 relevant articles out of 224, emphasizing studies employing impact forces to evaluate various aspects of dental implant treatments. The diverse applications of impact forces in dental research were categorized into tooth structure, restorative materials, interface evaluation, implant properties, and finite element models. Some studies showed the significance of impact forces in assessing stress distribution, shock absorption, and biomechanical response. Impact testing is a critical tool for understanding the daily forces on implants. Despite diverse experimental approaches, a lack of standardized protocols complicates the systematization of the results and, therefore, the conclusions. This review highlights the need for consistent methodologies in impact testing studies for future research on implant-supported prostheses.

## 1. Introduction

Dental implants have become an increasingly effective method to enhance the patient’s quality of life with single, partial, or total edentulism, as they can replace extracted teeth with restorations or prostheses over implants. The literature indicates that implant-supported prostheses are a reliable treatment with a favorable clinical success rate, although mechanical and biological complications can occur [1,2,3,4]. Many studies have been conducted to understand and address complications in implant prostheses, identifying factors that contribute to these difficulties and proposing solutions to reduce or even eliminate these complications. The most common complications that occur in implant treatments are related to mechanical factors, such as screw loosening; screw fractures; loss of the screw; loss of the screw’s resin covering; failure of metal, resin, or ceramic structure; and, in the case of overdentures, loss of retention [5,6].

Regarding the biological field, dehiscence is frequently observed in soft tissues around the implants after a sequence of several steps: inflammation, mucositis, bleeding, and suppuration. The main etiological agents that cause late failures are peri-implantitis and overload combined with host characteristics. High occlusal loads challenge implants, their components, and prostheses, potentially leading to mechanical failure and biological complications [1,7,8,9]. The combination of poor osseointegration of the implant, inadequate screw fixation of prosthetic components, and the exaggerated movements and dynamic forces involved can result in complex loading states, causing loosening and/or fracture of implant-supported components of prostheses. Prosthesis and implant design characteristics, along with the materials used and biomechanical considerations, have a significant impact on the long-term outcome of these types of rehabilitations [1,7]. Some studies and institutions, such as the well-known European Association for Osseointegration (EAO) [1,7,8,9], suggest that the application of excessive occlusal loads in a well-osseointegrated prosthesis may result in loss of marginal bone or even implant fracture, given that the aforementioned scenarios are related to peri-implant gingival inflammation. In the scientific literature, several tests assess the load applied to the implant, although most of these works were performed by using static loads, that is, they applied loads without movement on an immobile object [10,11,12,13,14,15,16,17,18,19,20,21,22]. However, there are very few studies testing dynamic forces. There are different types of dynamic force; for instance, incremental force is a constant force at a point, but unlike static forces, the value of force varies [23,24,25,26,27,28,29,30,31,32,33,34,35,36,37,38,39,40,41,42]. Another method, which is the main focus of this review, involves impact forces by applying a dynamic force on an immobile object. This approach is more closely related to the chewing force that implants experience in their daily function, forces caused by bruxism, or eccentric movements [36,43]. Regarding the numerical methods used to simulate the biomechanical response, there are different methods to compute the static forces, such as the Finite Element Method (FEM), which uses computational 3-dimensional models to analyze and evaluate the stress states in complicated structures, such as implants and the interface of prosthetic elements [44,45,46,47,48,49,50,51,52,53,54,55,56,57,58]. While prior investigations have predominantly focused on the examination of static loads on dental materials over an extended period, this review introduces a novel perspective. Specifically, it endeavors to employ a comprehensive literature review to elucidate studies that uniquely employ impact forces characterized by their transitory nature. The review aims to contribute to the scientific understanding of how dynamic loads, rather than static ones, influence and elucidate the properties of dental materials in the specific contexts of implant-supported prostheses and natural teeth.

## 2. Materials and Methods

**Figure 1:**
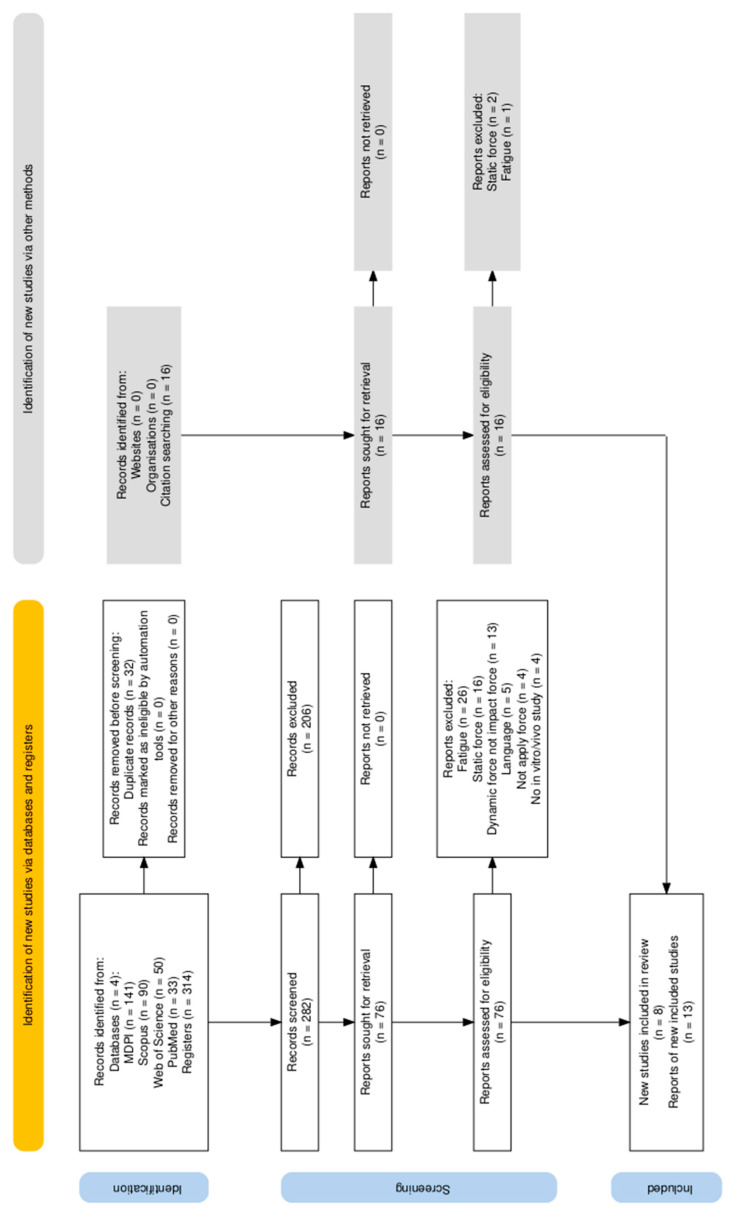
PRISMA flow diagram [60].

In this review, a systematic examination is conducted on the impact testing of fixed prostheses on implants and teeth. Twenty-one articles were included into this methodological review following the PRISMA protocol, as shown in Figure 1 [59,60].

The focused question was developed according to the PICO question (P = population, I = Intervention, C = Comparison, O = Outcome). The following PICO question was formulated: “In the context of implant-supported prostheses and natural teeth, how do impact loads influence the properties of dental materials compared to static and incremental loads in terms of durability and mechanical behavior?”. The subsequent sections will outline the process followed.

An extensive search was conducted across the PubMed, Web of Science, MDPI, and Scopus databases through the following research equations:

1. PubMed: ((Dental implants) OR (Rehabilitation Materials) OR (Restorative Materials)) AND ((Impact Load) OR (Dynamic Load) OR (Transient Load) OR (Dynamic Analysis)).
2. Web of Science: ((Dental implants OR Rehabilitation Materials OR Restorative Materials) AND (Impact Load OR Dynamic Load OR Transient Load OR Dynamic Analysis))
3. MDPI: ((Dental implants OR Rehabilitation Materials OR Restorative Materials) AND (Impact Load OR Dynamic Load OR Transient Load OR Dynamic Analysis))
4. Scopus: ((Dental implants OR Rehabilitation Materials OR Restorative Materials) AND (Impact Load OR Dynamic Load OR Transient Load OR Dynamic Analysis)).

Thirty-three results were provided by PubMed, 50 by Web of Science, 141 came from MDPI, and 90 results were found in Scopus, giving a total of 314 results. Before proceeding to study this selection, 32 duplicate results were eliminated and inclusion and exclusion criteria were established.

### 2.1. Criteria Used for Including Articles

- In vivo, in vitro, and computer modeling studies.
- There was no restriction regarding the properties that were evaluated using impact tests.

### 2.2. Criteria Used for Excluding Articles

- Studies that used static or incremental forces.
- Studies that did not analyze forces.
- Studies that used multiple impacts to determine the fatigue in implants.
- In vivo studies involving non-human animals.

### 2.3. Filters Used to Select Studies

- Studies conducted from 1991 to 2024.
- Studies only in English.

### 2.4. Studies Selection

Initially, the titles and abstracts of the results were evaluated, and 282 were deemed suitable, thus eliminating 206 during the process. Then, the 76 selected articles were downloaded.

Seventy-six studies were thoroughly reviewed, resulting in sixty-eight excluded articles. Specifically, 26 articles were discarded as they focused on analyzing the fatigue of different materials, 16 analyzed static forces, 13 analyzed dynamic forces that were not impact forces but rather incremental forces, 5 studies were published in languages other than English, 4 articles did not examine any forces on materials or teeth, and finally, 4 studies were not in vitro or in vivo studies. Finally, eight articles that satisfied the criteria in Section 2.1, Section 2.2 and Section 2.3 were considered in this review.

### 2.5. Manual Selection

After carefully reading the 8 articles and based on their bibliographic references, 16 articles not found in the databases using the established keyword combination were analyzed. Of these, upon further examination, 3 articles were discarded, 2 for analyzing static forces and 1 for studying material fatigue.

## 3. Results and Discussion

### 3.1. Tooth Structure

Impact forces can be used as a method for measuring properties in natural tooth structures. For instance, a remarkable study [61] determined the average impact compression stress causing fractures in central incisors by employing a drop ball impact test. This test consisted of two metallic bars inserted into a very rigid base in order to prevent displacement. The acceleration was measured through the use of stroboscopic photography to record the vertical drop of the ball. Furthermore, it is possible to measure and obtain relevant data from other parts of the mandible, such as the periodontal ligament (PDL), which helps in the PDL–bone–tooth stability. When the ligament is removed from an implant insertion, the dental bone structure is more prone to mechanical damage.

**Figure 2:**
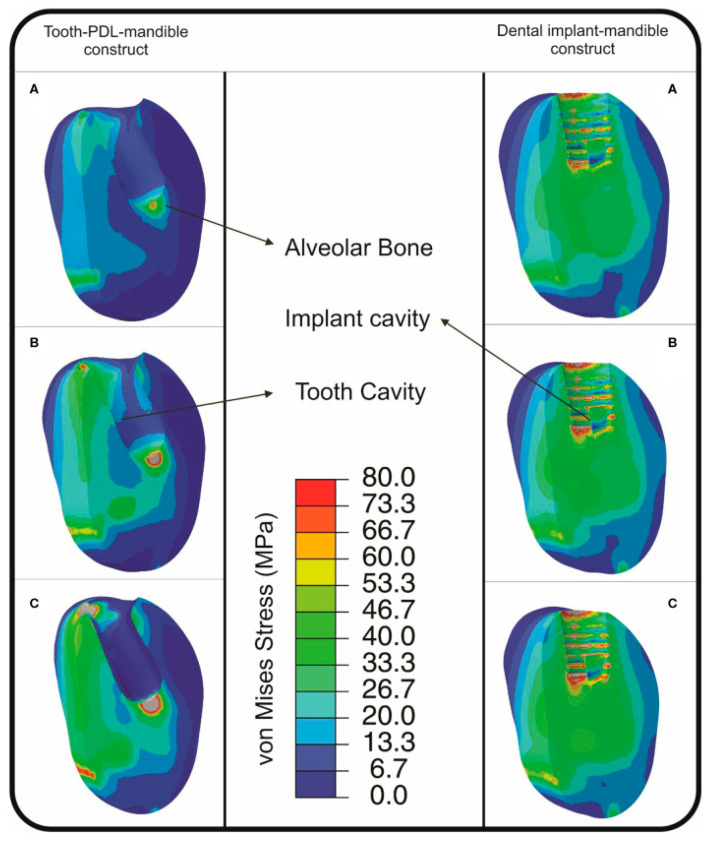
Von Mises stress distribution, found from FEM simulations, in alveolar bone in tooth–PDL–mandible (left: A, B, C) and dental implant–mandible (right: A, B, C) construct, resulted from impact loading simulations for the release heights of: (A) 1 cm; (B) 2 cm; and (C) 3 cm in the study [62].

The authors of another interesting investigation [62] compared how much mechanical impulse was transmitted to the model components (periodontal ligament, bone, tooth, and implant) during the impact (see Figure 2). They began by performing impact tests on the jaws of dogs, with and without dental implants, using a special drop tower device. This device allowed them to vary the fall’s height between 1 and 3 cm and, therefore, vary the impact load. Their experiments showed that, at each height (1, 2 and 3 cm), the model received 33.1%, 31.0%, and 27.5% more momentum than the tooth–ligament–bone structure, respectively. Additionally, FEM computational simulations confirmed these experimental findings, showing errors of less than 7.5%.

### 3.2. Restorative Materials

**Figure 3:**
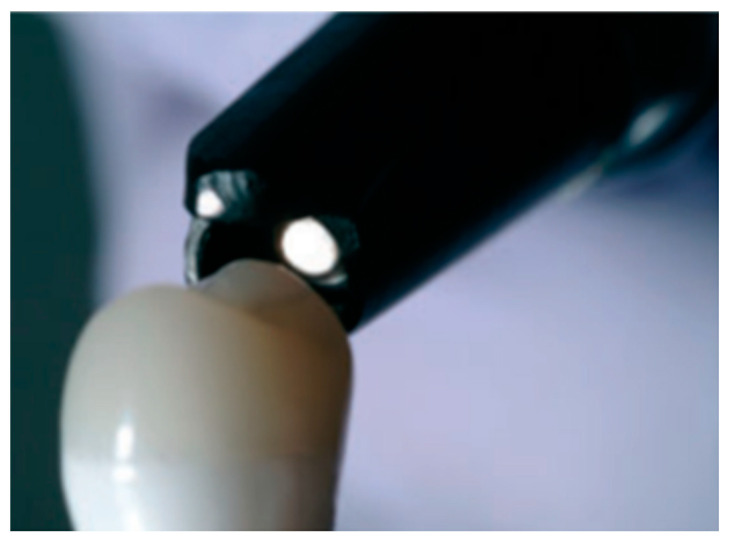
The periometer and its probe with percussion rod. Probe pulled back to show percus-sion rod-that was used in the study [64].

Restorative dental materials are defined as those utilized for the rehabilitation of damaged teeth and the replacement of missing teeth. Impact forces are used to measure the capacity of rehabilitation materials to absorb impacts. A relevant study [43] designed a test apparatus consisting of an inclined platform with an included groove at the end of which a force transducer was mounted on an acrylic resin block. Another interesting investigation [63] use a test apparatus that consisted of a hammer connected to an oscillating hammer. Another relevant work [64] used the Periometer as shown in Figure 3. Thanks to the percussion it generated, the authors were able to evaluate the coefficient of lost energy and the return of force of both dental and implant restorations.

Second, impact testing was used to measure the ability of fractured teeth reinserted with restorative materials to withstand impact. An in vitro study [65] utilized a well-known testing machine (Instron, MA, EEUU) to investigate how different materials and reinsertion methods behave regarding the impact resistance capacity of bovine incisor teeth. The study concluded that neither technique nor material, when considered individually, could reach the mechanical resistance of healthy teeth.

By using a pendulum-type testing machine [66], the authors concluded that restoring fractured teeth with fragment reattachment using 3M Single Bond and 3MZ ‘100’ (resin made from various materials) resulted in impact resistance comparable to that of untouched teeth. Two other studies [3,67] used a masticatory robot, which simulated the physiological human masticatory cycle, to observe the effect that occlusal loads can have on restorative materials.

### 3.3. Bone–Implant Interface

Impact forces are also used to evaluate the response of the bone–implant interface. For instance, the relevance that the quality of bone has on the stress levels by loading two implant-bone mimicking models with impact forces was investigated in [68]. The experiment consisted of dropping a metal rod, simulating an impact.

Another interesting study [69] designed a modal dynamic-based testing method that was able to non-invasively evaluate the interface of an endosseous prosthesis subjected to a side impact from an impedance-headed hammer. The dynamic modal-type test was capable of differentiating between interfaces based on the bone-tissue type and the level of fixation existing between the interface and the implant.

### 3.4. Implant Properties

Many implant properties are evaluated using impact forces. A good example can be found in [70]. The authors demonstrated the discrepancy, in terms of resilience, between acrylic-based resin and ceramic-based materials used for coating, although this difference can only be measured through an in vitro experiment where the load is produced by an impact and the implant is fully fixed.

A strain-gauged abutment was employed to evaluate the load transmitted to the implant following the impact. A pendulum-based method, such as that proposed by Charpy, was used with a 3.95 J-force to compute the effect of microwave polymerization cycles on resistance to impact and the type of fracture of the acrylic-based resin used in the base of dentures, as reported in [71]. The researchers concluded that the impact resistance of acrylic-based resins subjected to microwave polymerization changed depending on the irradiation period.

**Figure 4:**
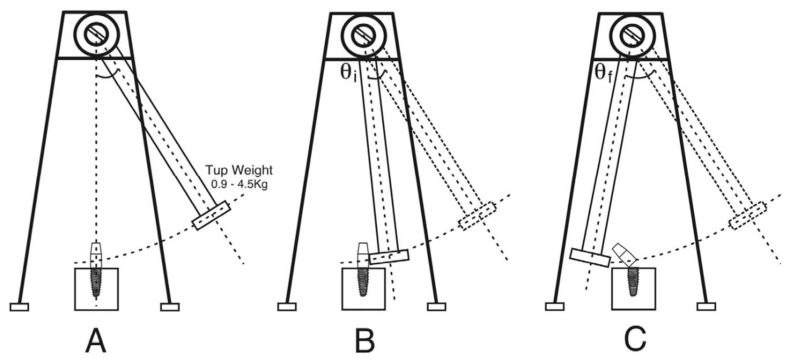
The pendulum-based impact test device that was used in the study [72] of the impact test performed in this investigation. (**A**) Shows the test setup and its release angle. (**B**) Shows the point of impact and the angle between the release point and the point of impact. (**C**) Shows the implant fractured after impact and the angle between the release point and maximum follow-through [72].

In contrast, the fracture resistance of both titanium and ceramic endosseous oral implants subjected to impact loads has also been analyzed [72]. The implants were tested in two distinct mounting devices, brass and a material that emulated the bone. The testing apparatus used was a pendulum device with variable weights starting at 0.9 and going up to 4.5 kg (see Figure 4). The findings indicated that both the implant and abutment made of titanium required the highest energy to cause the failure of the implant–abutment assembly when held in brass, while the ceramic abutment on a titanium implant exhibited the lowest energy to produce the failure. However, no relevant differences were noted when the assemblies were inserted into bone substitute foam bricks. These results indicate that the energy required to fracture both the titanium and ceramic implant assemblies is actually very low when using the elastic modulus of bone substitute foam bricks. However, greater differences were observed when increasing the elastic modulus of the material where the system is inserted.

### 3.5. Finite Element Analysis (FEA) Models

Several studies using modeling and computer simulation have evaluated how stresses are distributed in both the implant and the surrounding bone. A research study [73] used a three-dimensional finite element model to progressively apply static and dynamic occlusal forces on an implant-supported model covered with GC GRADIA porcelain and resin in a human mandible. The study revealed that greater stresses at the interface between the bone and the implant were observed in the area of cortical bone closest to the implant’s initial thread in all simulations. The models with static loads at the implant exhibited higher stresses and strains than those subjected to dynamic loads. The direction of the load was an important variable when calculating the stress distributions, with variations that reached 85% in some cases. It was observed that GC GRADIA resin could reduce the impact load up to values of 6.5%. The study indicated that the influence of the implant superstructure and covering materials on both strains and stresses at an implant-supported model is only slightly significant, showing differences around 1%.

Another related study [74] proposed an optimal dental implant with regard to both stress, displacements, and strain distributions on the neighboring cortical and trabecular bones. The study sought to evaluate the dynamic, fatigue, and static responses of implants in order to gain a comprehensive understanding of the underlying mechanisms. Three different configurations of a tapered dental implant were studied: V-shaped threads (model 1); V-shaped threads in the body implant and microthreads in the upper area (model 2); and reverse buttress threads in the whole-body implant (model 3). Two different forces were applied to a finite element 3D model, with the resulting stress and strain distributions in the supporting bone being evaluated. The study demonstrated that the lowest von Mises stresses on cortical bone were observed in model 2. This value was 44.5 MPa in the static analysis, while a value of 47.4 MPa was recorded in dynamic analysis, both of them produced by a 100 N applied load and inclined 25° with respect to the vertical direction. The authors concluded that the best combination to obtain a uniform distribution of stresses in a conical implant was achieved with a V-shaped thread throughout the whole implant and microthreads in the upper region. The simulations were static and dynamic.

A FEM analysis of implants with impact loading, focusing on the influence of the implant’s geometry and thread type, was reported in the literature [75]. This study was conducted on a 3D bone model obtained from a cone–beam computerized tomography (CT) scan of a patient. The implant geometries were generated using the widely recognized SolidWorks software [76]. The load was simulated using an explicit dynamic approach by striking a solid body with a 1 mm/s speed against the implant in both the vertical and horizontal direction. The stress magnitude and distribution generated on both cortical and cancellous bone were obtained by using the well-known ANSYS software [77]. The numerical analysis demonstrated that the highest stresses appeared in cortical bone in group number 1 (straight threads), while the highest stress in the cancellous bone and implant was observed in the second group (triangle-shaped threads). The researchers concluded that implants with deeper threads showed better stability, probably due to the increase in the contact surface between the implant and the bone. In contrast, implants with smaller thread sizes and shorter pitch-lengths produced greater bone stresses.

The assessment of the dynamic behavior of implants with stress-absorbing elements has been also reported in technical literature [78]. Two models were used: one with a stress-absorbing element made of polyoxymethylene (experimental group) and another with rigid titanium (control group). Analyses performed using the FEM showed that the tested group exhibited a lower natural frequency and greater damping when the results were contrasted with those of the control group. This research demonstrated the importance of understanding the responses of impact loadings for a better comprehension of the implant’s stability in oral conditions.

Another work [79] investigated how the implant threads and the direction of occlusal loading can affect both the mechanical and biological response of the implant and the supporting bone using FEA simulations. The study analyzed shear, compression, and tensile stresses, as well as von Mises stresses in the implant and the adjacent bone tissue, performing static, dynamic, and quasi-static simulations. Strains were also considered. The outcomes demonstrated a notable impact of both thread configuration and occlusal loads on the stresses and strain levels in realistic clinical environments.

**Figure 5:**
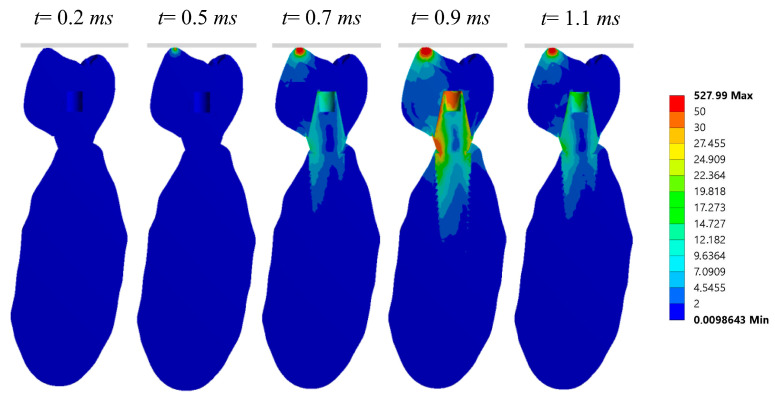
Von Mises stress distribution over time at a cross section using the Carbon Fiber-Ceramic crown, considering both trabecular and cortical bone [46].

The biomechanical behavior of dental rehabilitation models when using different materials for the crown and considering whether or not cortical bone exists can be found in [46] (see Figure 5).

This research considered six different materials for crown rehabilitation in FEM modeling: all metal, metal and composite, metal and ceramic, composite of PEEK, composite of carbon fiber, and carbon fiber with ceramic. A dynamic analysis using the FEM was conducted on models of the aforementioned crowns to evaluate their biomechanical responses when subjected to dynamic loading. Implant crown specimens with the presence (or absence) of cortical bone were investigated to evaluate the effects of impact forces on both types of scenarios. The findings indicated that less rigid (more flexible) restorative materials contributed to the reduction in stress in both bone tissues (cortical and cancellous). Consequently, they would be particularly recommended in situations with little presence of cortical bone. Furthermore, the study revealed that the use of stiffer materials (e.g., metals and ceramics) in implant-supported restorations results in higher stresses at the cortical and trabecular bone, while the use of less rigid (more flexible) materials results in a reduced transfer of stresses. Another significant result in implant-supported cases was that the stresses caused by dynamic loading in the bone were higher when there was a lack of cortical bone.

**Figure 6:**
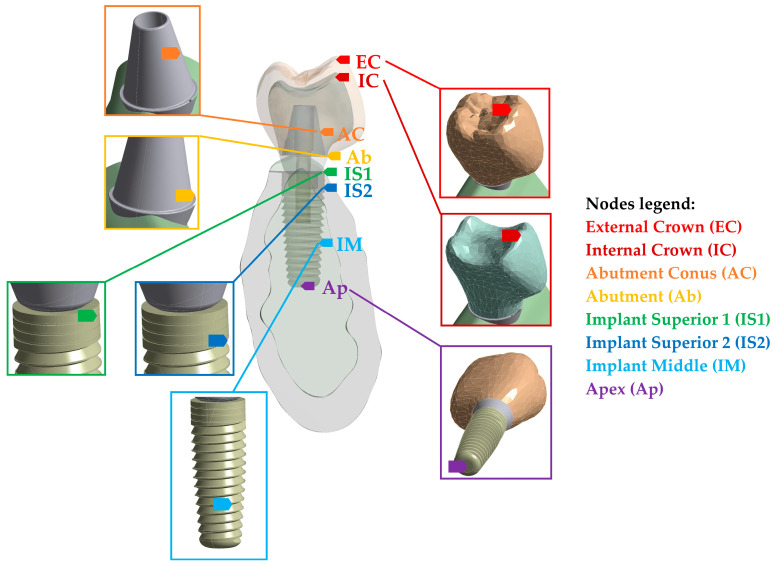
The eight reference nodes used for numerical simulation in [47].

Another recent investigation [47] identified the optimal mechanical behavior of rehabilitation materials in a single implant-supported prosthesis subjected to impact forces. This involved the testing of distinct material combinations for the inner crown and the outer one. The analyses focused on the dynamic stresses obtained at eight distinct regions of the implant (see Figure 6) and evaluated the effects of mechanical properties, including the elastic modulus, the Poisson’s coefficient, the material’s density, and the initial conditions, in terms of speed. A complete three-dimensional model was used, which included the crown, the retention screw, the implant, and the involved part of the mandible. The study employed the FEM to evaluate the repercussions that different restorative material combinations had on the implant’s stresses. The research findings underscore the significance of restorative material choice in preserving the osseointegration and highlight the impact of an inadequate Young’s modulus on implant survival.

**Figure 7:**
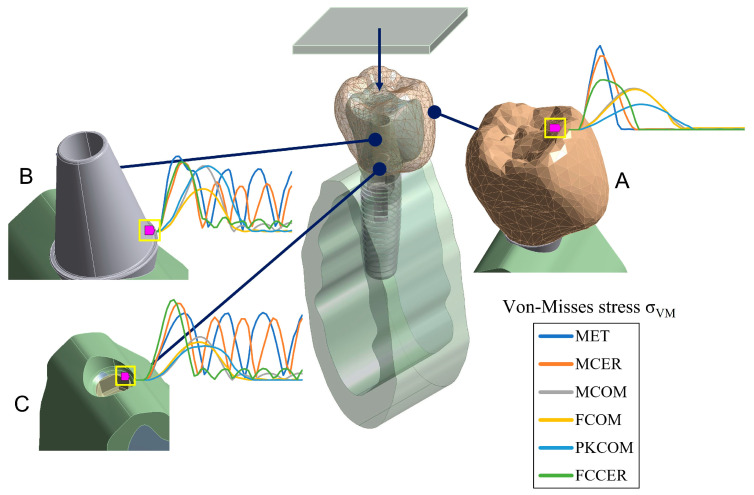
The FEM model and the node selected for numerical simulation in the study cited in [44]. (**A**) Sectional view of the 3D FEA model at the crown node. (**B**) The abutment node. (**C**) The node on top of the cortical bone.

The stresses produced by dynamic impact loads acting on an implant-supported model using 3D FEA has been also investigated [44]. A three-dimensional model of an implant-supported prosthesis inserted into a portion of the human mandible was configured using computer-aided design (CAD) and reverse engineering. Six different implant-supported models were constructed from a variety of material combinations, which included metal, composite, ceramic, PEEK, and carbon fiber. A full 3D dynamic analysis using the FEM was performed to model the impact of an 8.62 g implant-supported device against a solid plate at a 1 m/s speed after a 0.01 mm displacement as shown in Figure 7. The study calculated the maximum stress transmitted to all the device components (crown, abutment, implant, and cortical bone tissue), as well as the evolution of stresses over time.

In numerical studies on dental implantology, the model characteristics and material properties can significantly impact results, presenting important limitations. One of the primary sources of uncertainty stems from the assumption of values for the tissues, as these can vary considerably in reality. Additionally, many studies in this review modeled only a portion of dental substructures and considered them as isotropic and homogeneous, despite the fact that no anatomical dental structure is truly isotropic or homogeneous [49,50]. This simplification can lead to results that do not accurately reflect the actual behavior of dental structures under impact conditions. The lack of consideration of the anisotropic and heterogeneous properties of dental tissues limits the ability of models to precisely predict the responses of implants and surrounding tissues to impact loads, highlighting the need for a more detailed and realistic approach in future studies.

### 3.6. Prospect Section

The study of impact forces applied to implants presents a promising approach to improving the clinical success of implants, abutments, and the prostheses placed on them. Additionally, alongside the study of both innovative and commonly used materials for implant-supported prostheses, it could help us to understand and reduce the risk of complications in this field. Future research should, firstly, aim to standardize protocols for studying impact forces using the various existing methodologies, and, secondly, extend the study to different implant-supported prostheses and scenarios, as most studies focus solely on single crowns on implants, applying an impact force with a rigid body. For example, the distribution of forces in a prosthesis supported by two or more implants or the impact produced by the collision of two implant-supported prostheses against each other could be investigated.

## 4. Conclusions

This systematic review highlights that impact testing, despite its significance in mimicking the day-to-day forces experienced by dental implants, remains less prevalent in the literature compared to fatigue, static, and incremental testing methods. The impacts, which implants encounter every day, carry the potential to induce both mechanical and biological complications. The review underscores the fact that various researchers have drawn noteworthy conclusions by incorporating impact testing into their investigations. Impact testing is vital in assessing the mechanical properties of natural teeth and implant-supported prostheses by assessing the forces they experience in real life. The review underscores that various researchers have drawn noteworthy conclusions by incorporating impact testing into their investigations. However, it should be remarked herein that most impact studies involve simulations, with limited in vitro or in vivo literature regarding impact tests. Increasing the in vitro and in vivo approach would lead to a more realistic understanding of these forces by analyzing research outcomes more closely with real-world scenarios.

For natural teeth, such testing helps to determine fracture resistance and highlights the protective role of the periodontal ligament (PDL) in absorbing shock and distributing stress, thus maintaining the integrity of the tooth–bone structure.

In implant-supported prostheses, impact testing has revealed that implants can transmit higher forces to the surrounding bone compared to natural teeth, potentially increasing stress and risk of damage. The impacts in dental implants carry the potential to induce both mechanical and biological complications. Impact testing also evaluates how restorative materials like resins and ceramics absorb impact, affecting stress distribution and long-term durability.

Additionally, a noteworthy challenge emerges from the observed diversity in experimental approaches across the reviewed studies. Despite utilizing impact testing, the absence of a standardized or common experimental protocol introduces complexity into the process of synthesizing conclusions from multiple studies. The studies use different variables for analysis, such as Newtons, Joules, megapascals, or grams. They also utilize different methodologies, such as dropping a weight, using a pendulum, or throwing masses of varying magnitudes at different distances and speeds. All of these aspects are part of the diversity of approaches in the studies.

Standardization is crucial in dental implant impact testing for producing realistic and comparable results, which are necessary for accurately evaluating the stability and performance of various materials and implant designs. The current lack of uniformity in test methods and magnitudes presents a significant challenge in terms of drawing generalizable insights. By implementing standardization in the testing protocols, researchers can address potential challenges and improve the reliability of results in the field of dental implant research.

## Data Availability

Not applicable.
